# Transparent sparse graph pathway network for analyzing the internal relationship of lung cancer

**DOI:** 10.3389/fgene.2024.1437174

**Published:** 2024-10-01

**Authors:** Zhibin Jin, Yuhu Shi, Lili Zhou

**Affiliations:** ^1^ Information Engineering College, Shanghai Maritime University, pudong, China; ^2^ Yangpu District Central Hospital, Shanghai, China

**Keywords:** graph neural networks, biological pathway, edge prediction, ceRNA, LUSC

## Abstract

While it is important to find the key biomarkers and improve the accuracy of disease models, it is equally important to understand their interaction relationships. In this study, a transparent sparse graph pathway network (TSGPN) is proposed based on the structure of graph neural networks. This network simulates the action of genes *in vivo*, adds to prior knowledge, and improves the model’s accuracy. First, the graph connection was constructed according to protein–protein interaction networks and competing endogenous RNA (ceRNA) networks, from which some noise or unimportant connections were spontaneously removed based on the graph attention mechanism and hard concrete estimation. This realized the reconstruction of the ceRNA network representing the influence of other genes in the disease on mRNA. Next, the gene-based interpretation was transformed into a pathway-based interpretation based on the pathway database, and the hidden layer was added to realize the high-dimensional analysis of the pathway. Finally, the experimental results showed that the proposed TSGPN method is superior to other comparison methods in F1 score and AUC, and more importantly, it can effectively display the role of genes. Through data analysis applied to lung cancer prognosis, ten pathways related to LUSC prognosis were found, as well as the key biomarkers closely related to these pathways, such as HOXA10, hsa-mir-182, and LINC02544. The relationship between them was also reconstructed to better explain the internal mechanism of the disease.

## 1 Introduction

Lung cancer is one of the most common malignant tumors in the world and is related to high morbidity and mortality (Siegel et al., 2021). Approximately 85% of patients suffer from non-small cell lung cancer (NSCLC), of which lung squamous cell carcinoma (LUSC) and lung adenocarcinoma (LUAD) are the two most common subtypes (Herbst et al., 2018). In addition, because lung cancer has a complex molecular mechanism, it is a heterogeneous disease which involves a complex interaction between genes and the environment. Therefore, targeted therapy may not be effective for patients, and it leads to significant differences in the prognosis of patients with the same type of cancer (Dagogo-Jack and Alice, 2018). Therefore, it is necessary to develop a more accurate method based on the internal mechanism of molecular features for the research and analysis of lung cancer and to mine effective biomarkers from it. This is very important for individual treatment decisions and targeted treatment.

Because of the complexity of lung cancer, a multi-omics methodology will be more beneficial for capturing the potential molecular correlation and key genes in lung cancer. Among these, the ceRNA network embodies this well, including for mRNA, miRNA, and lncRNA. It not only contains a variety of data with their interactions but also imitates the regulation relationship between RNAs and makes the results more biologically interpretable (Salmena et al., 2011). Using the ceRNA network as prior knowledge or a premise, the information obtained will be more accurate than using single datum. For example, Li et al. (2021) annotated DE lncRNAs and mRNAs through gene ontology (GO) and Kyoto Encyclopedia of Genes and Genomes (KEGG) pathway analysis, and obtained ceRNA networks related to prognosis by using bioinformatics analysis tools such as Kaplan–Meier (KM) survival analysis and the LUAD database in the Cancer Genome Atlas (TCGA); they finally identified potential biomarkers.

However, traditional statistical or bioinformatics analysis needs to be adapted to different cancer types, and such methods still have some limitations in high-dimensional analysis and screening representative features. The application of graph neural networks (GNNs) in the field of bioinformatics has been a popular in recent years; it can effectively construct protein–drug or protein–protein interaction (PPI) networks and has good explanatory power. Therefore, it is feasible to obtain the interaction relationship between genes through GNNs. For example, Bastian et al. (2022) obtained an explainable GNN connection through the disease subnetwork detection algorithm, which determines the connection between genes using PPI combined with multi-omics data such as gene expression data and methylation data. A community with the highest score, or sub-network, is then calculated by the community detection algorithm according to the obtained edge weights. However, the choice of its community still needs the help of existing connections, and it is impossible to predict the possible relationship. Kang et al. (2022) proposed a GNN based on link representation to predict molecular association, which obtained gene embeddings through an encoder combined with a biological network to reconstruct the network through a decoder while the network is still a black box model and its explanation is not good.

Therefore, in order to improve the biological interpretability of the model, biological pathway data should be integrated into the network. This can not only incorporate the existing biological knowledge into the model but also can determine the internal biological processes involving the pathways and the corresponding genes and protein (Jin et al., 2014; Kim et al., 2012). It can also explain the experimental results based on the pathway, which has a more intuitive and comprehensive understanding of the molecular mechanism related to function. For example, Elmarakeby et al. (2021) developed a P-NET model to discover key genes related to the prostate, constructing a progressive network model using the existing biological knowledge and combining multi-omics-related information to realize a network with internal nodes giving complete transparency and knowledge. However, its construction needs the help of biological knowledge and experiments which may not be obtainable or possible.

In order to solve these issues, we here propose a transparent sparse graph pathway network (TSGPN) model based on a GNN. First, the network connection was initialized according to a ceRNA network, PPI network, and multi-omics information, and the final trend of the network was set as mRNA—the final influence of other genes on mRNA. Second, the mRNA was connected to its corresponding pathway to form a pathway neural network according to the pathway database so that the gene could be effectively explained by the corresponding pathway. Finally, the hard concrete estimation algorithm was used to continuously remove the interfering connections in the initialized network to predict the connection during the iterative process, leaving sparse and obvious key biomarkers as well as their interactions.

## 2 Materials and methods

**FIGURE 1:**
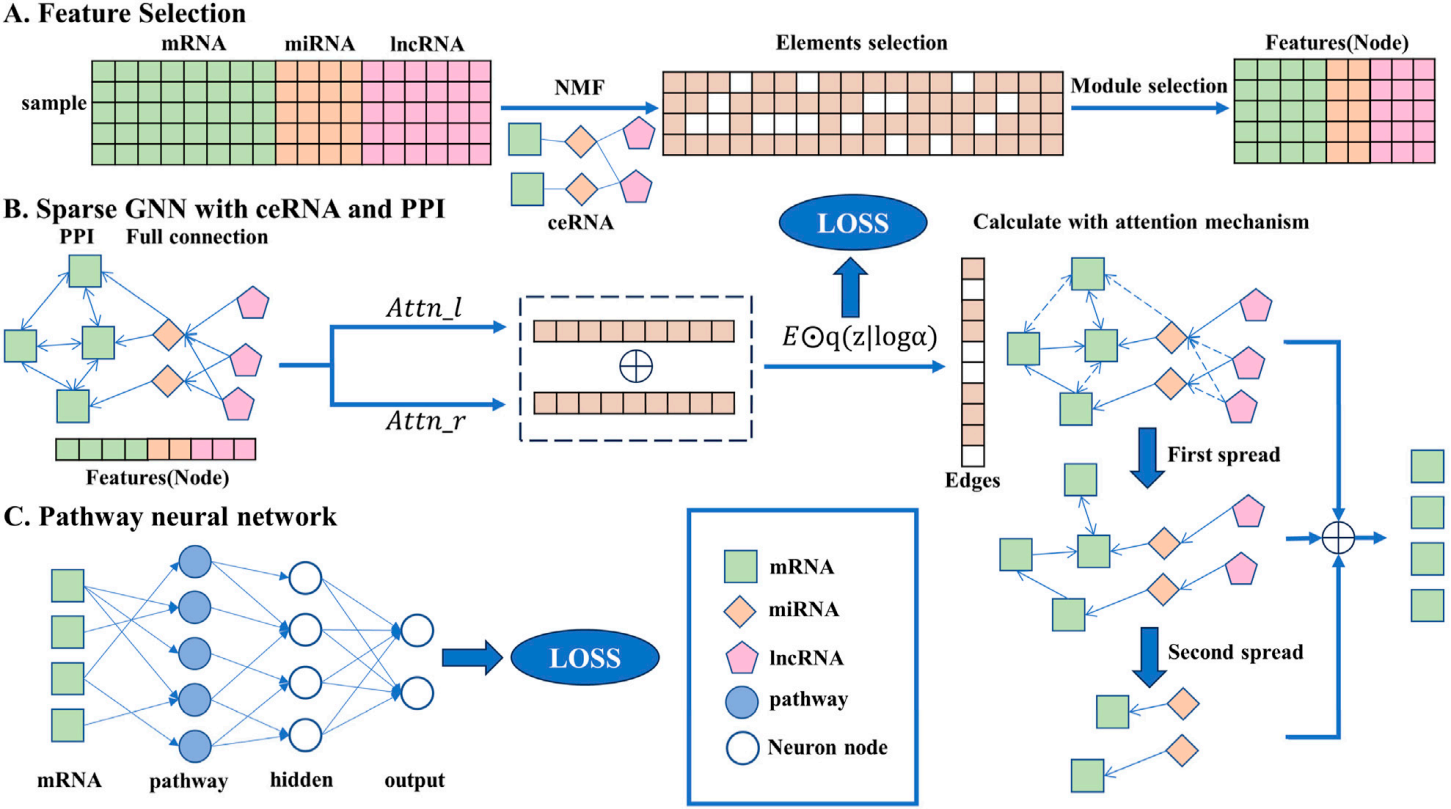
Overall flowchart of the transparent sparse graph pathway network, the end of each step corresponding to the beginning of the next. In A, the key biomarkers are obtained from the original data through the NMF algorithm, element selection, and module selection operations as the input of the model. In B, all the key biomarkers will be fully connected, the interaction between each gene can be obtained through side screening, and the affected mRNA can be obtained through simulated propagation. In C, the mRNA is trained by a pathway neural network, and the result is output.

In this section, the detailed method descriptions are given and the overall flowchart is shown in Figure 1, including the data preprocessing stage, network construction, and iterative rules.

### 2.1 Feature selection

Bio-interpretability requires that the output of the algorithm is sparse, which also shows that only a few genes play a key role in the disease. Therefore, the feature selection of the input data should not only be consistent with biological characteristics but also help improve the accuracy and speed of the algorithm.

In this study, the improved non-negative matrix factorization (NMF) algorithm was used to extract features from the input data; this is an effective analysis method for processing large-scale data, and it is also currently the mainstream decomposition algorithm in biology, with good biological interpretability. Compared with traditional algorithms, it has obvious advantages in simplicity, decomposition form, and interpretability of decomposition results (Paatero and Tapper, 1994).

In order to pay more attention to the correlation between data in decomposition, the interaction matrix of the ceRNA network was added as prior knowledge, including mRNA-miRNA and miRNA-lncRNA, in which the up-/downregulation information of genes obtained in the differential analysis stage was used to add weight to these correlations. In particular, we used Equation 1 to calculate the values in the interaction matrix; only the connected relationship existing in the ceRNA network was calculated.

$$a_{ij},b_{ij}=\left\{\begin{array}{c} -e^{-\left|\left|{r1}_{i}\right|-\left|{r2}_{j}\right|\right|},if\;{r1}_{i}\;*\;{r2}_{j}<0,\\ e^{-\left|\left|{r1}_{i}\right|-\left|{r2}_{j}\right|\right|},if\;{r1}_{i}\;*\;{r2}_{j}>0 \end{array}\right.$$ (1)

where $a_{ij}$ represents the elements in interaction matrix A between mRNA and miRNA, and $b_{ij}$ represents the elements in interaction matrix B between lncRNA and miRNA. They are in a competitive relationship in the ceNRA network. mRNA and lncRNA will give miRNA. ${r1}_{i}$ and ${r2}_{j}$ represent the logFC values calculated by DEseq2 (Love et al., 2014) on the *i* ^th^ and *j* ^th^ gene.

In addition, the kernel function method was used to map the data into high-dimensional space so as to find some potential key genes. Based on the kernel joint non-negative matrix factorization proposed by Salazar et al. (2021), each kind of data $X_{I}$ is mapped into the high-dimensional space $\emptyset \left(X_{I}\right)$ . The kernel function $k_{IJ}=e^{-\left(\frac{{\left\|X_{i}^{I}-X_{j}^{J}\right\|}^{2}}{2\sigma }\right)}$ is used to represent the distance between these two kinds of data, in which I and J represent two different kinds of data, i and j represent the column of matrix, and σ is used to set the control range of the kernel function. The objective function is given in Equation 2 as follows:

$$\begin{array}{ll} \tau \left(Q_{1},Q_{2},Q_{3},H_{1},H_{2},H_{3}\right) & =\min\left(\sum_{I=1}^{3}\left(Tr\left(k_{I}-k_{I}Q_{I}H_{I}-H_{I}^{T}Q_{I}^{T}k_{I}+H_{I}^{T}Q_{I}^{T}k_{I}Q_{I}H_{I}\right)+\alpha \left({\left\|H_{I}H_{I}^{T}-I_{I}\right\|}_{F}^{2}\right)\right)\right)\\ & -\lambda_{1}Tr\left(H_{2}AH_{1}^{T}\right)-\lambda_{2}Tr\left(H_{2}BH_{3}^{T}\right)+r_{1}\sum_{I=1}^{3}{\left\|Q_{I}\right\|}_{F}^{2}+r_{2}\sum_{I=1}^{3}{\left\|H_{I}\right\|}_{1}+w\left(Q_{1}^{T}k_{1}Q_{1}-Q_{1}^{T}k_{12}Q_{2}-Q_{2}^{T}k_{21}Q_{1}+Q_{2}^{T}k_{2}Q_{2}\right)\\ & +w\left(Q_{1}^{T}k_{1}Q_{1}-Q_{1}^{T}k_{13}Q_{3}-Q_{3}^{T}k_{31}Q_{1}+Q_{3}^{T}k_{3}Q_{3}\right)+w\left(Q_{2}^{T}k_{2}Q_{2}-Q_{2}^{T}k_{23}Q_{3}-Q_{3}^{T}k_{32}Q_{2}+Q_{3}^{T}k_{3}Q_{3}\right), \end{array}$$ (2)

where $k_{IJ}=\emptyset \left(X_{I}\right)\emptyset \left(X_{J}\right)$ , and $k_{I}$ is the abbreviation for $k_{II}$ . The origin matrix $k_{I}$ is decomposed into $k_{I}Q_{I}H_{I}$ , and w is used to control the similarity between $\emptyset \left(X_{I}\right)Q_{I}$ matrices in which the iterative formulas of $Q_{I}$ and $H_{I}$ can be obtained according to the Lagrange multiplier method and KKT conditions.

Next, the module with the highest correlation was selected by disease enrichment analysis, and the module elements were selected by the method proposed by Deng et al. (2021). The Z-score transformation was used for the method expressed as $Z_{ij}=\left(h_{ij}-\mu_{i}\right)/\sigma_{i}$ , where $h_{ij}$ is the element value in each module, $\mu_{i}$ is the mean value of each module, and $\sigma_{i}$ is the standard deviation of each module. For each element $h_{ij}$ , if its Z-score value is greater than the threshold T , this element is considered a prominent feature in this module.

Finally, using the K value of 42, module 5 was selected as the feature selection module, and 88 mRNA, 19 miRNA, and 208 lncRNA in this module were obtained. They were spliced into a large matrix X which was used as the input of the subsequent algorithm.

### 2.2 Sparse graph neural network with ceRNA and PPI

Although key genes can be obtained by feature selection, the relationship between them and the mechanism of their mutual operation are not very clear. In the previous method, the relationship was expressed by calculating the Spearman correlation between genes. In this study, a fully connected network of mRNA–miRNA and miRNA–lncRNA will be established in the initial state by constructing a GNN, and the redundant links will be removed through gradual iteration so as to rebuild the ceRNA network. Based on the method proposed by Ye and Ji (2023), we added the ceRNA and PPI networks to improve and consider their characteristics. The network was then spread twice to conform to the mechanism of ceRNA.

In order to realize the edge removal operation, a binary parameter was attached to each edge to determine whether to reserve this edge for neighbor aggregation, these removed edges being noise data or unimportant edges.

$$\dot{E}=E⊙Z,Z\in {\left\{0,1\right\}}^{M},$$ (3)

where E represents all edges in graph G, including the PPI, ceRNA, and gene full connection networks, and M is the number of edges in graph G.

Based on the principle of a graph convolution network (GCN) (Kipf and Welling, 2017) and following the neighbor clustering mechanism, the low-level features of nodes and their neighbors was aggregated, generating a new high-level feature representation to realize feature extraction. Its encoder function is expressed in Equation 4 as follows:

$$f\left(X,E,W\right)=\sigma \left(\hat{E}XW\right),$$ (4)

where σ is the activation function and *W* is the weight of the GCN network. $\hat{E}={\hat{D}}^{-1/2}\hat{E}{\hat{D}}^{-1/2}$ , and ${\hat{D}}_{ii}=\sum_{j}{\hat{E}}_{ij}$ . It can also be expressed in Equation 5 as

$$h_{i}^{\left(l+1\right)}=\sigma \left(\sum_{j\in N_{i}}{\hat{E}}_{ij}h_{j}^{\left(l\right)}W^{\left(l\right)}\right),$$ (5)

where l represents the number of layers. $h_{i}^{\left(l+1\right)}$ represents the hidden value of node *i* at layer l+1 , and $h^{\left(0\right)}=X$ . $N_{i}$ denotes all the neighbors of node *i* not including itself.

Because the association strength between different genes differs, a graph attention network (GAN) (Velikovi et al., 2017) was added to the algorithm to find the most influential connection; this has achieved good results in many neural network tasks (Xu et al., 2015; Bahdanau et al.). The attention mechanism can give different weights to the edges so that the algorithm can pay more attention to the edges with larger weights and extract more significant features.

Therefore, it is necessary to define a value for each edge. Because the relationship between genes is determined by genes, the value of an edge can be determined by the two vertices connected by this edge. The value of each edge is defined as the sum of the values of its two vertices as shown in Equation 6.

$$E_{ij}=X_{N_{i}}+X_{N_{j,}}$$ (6)

where $X_{N_{i}}$ represents the feature value when the node is *i*. In order to control the value of the edge between 0 and 1, each edge is normalized in Equation 7 as follows:

$$e_{ij}=normalize\left(E_{ij},Z_{ij}\right)=\frac{E_{ij}Z_{ij}}{E_{kj}Z_{kj}.}$$ (7)

The edge value is only calculated at the beginning of each training, and the subsequent convolution operation only uses the edge value at the first calculation. Therefore, after passing through the GNN layer and secondary convolution, the final value is obtained by Equation 8

$$h^{\left(3\right)}=h^{\left(0\right)}+h^{\left(1\right)}+h^{\left(2\right)},$$ (8)

where $h^{\left(3\right)}$ is used in the following pathway neural network (PNN), in which only the involved mRNA will be preserved.

### 2.3 Gradient estimation of discrete value Z

Because Z is a binary mask, its value is not differentiable, so it is necessary to use an approximate algorithm to solve this problem. The hard concrete gradient estimator is an algorithm with good effect and relatively simple implementation. It uses a reparameterization trick to approximate Equation 3 by a close surrogate function in Equation 9:

$$\dot{E}=E_{u∼U\left(0,1\right)}\left(E⊙g(\right.\sigma \left(\left(log\;u-log\;\left(1-u\right)+log\;\alpha \right)/\beta \right)\left(\zeta -\gamma \right)+\gamma ))$$

$$g\left(\cdot \right)=\min\left(\max\left(0,\cdot \right),1\right)$$

$$\log\;\alpha ={attn\_l}_{N_{i}}\cdot X_{N_{i}}+attn\_r_{Nj}\cdot X_{N_{j}},$$ (9)

where attn_l and attn_r are the learning parameters, and β=2/3,γ=−0.1,ζ=1.1 . These are the typical parameter values of the hard concrete distribution, more details of which can be found in Louizos et al. (2018). $U\left(0,1\right)$ is a uniform distribution in the range of [0,1]. σ is the activation function $\sigma \left(x\right)=\frac{1}{1+e^{-x}}$ . In the testing stage, the above formula changes Equation 10 as follows:

$$\dot{E}=\left(E⊙\sigma \left(\log\;\alpha /\beta \right)\left(\zeta -\gamma \right)+\gamma \right).$$ (10)

Due to the hard concrete gradient estimator, the binary parameter Z changes to a continuous value from 0 to 1, and most of the edges will be deleted to form a sparse network through iteration. In addition, the loss of the GNN in Equation 11 needs to be calculated for optimization as follows:

$${loss}_{GNN}=\lambda_{1}\sum_{\left(i,j\right)\epsilon N}\sigma \left(\log\;\alpha_{ij}-\beta \log\;\frac{-\gamma }{\zeta }\right),$$ (11)

where $\lambda_{1}$ is a regularization hyperparameter that controls the degree of edge sparseness.

### 2.4 Pathway neural network

Similar to a GNN, a PNN is also a simulation of a biological process, providing the biological interpretability of a neural network. It consists of an input layer (representing genes), a biological pathway layer associated with genes, a hidden layer of the relationship between biological pathways, and an output layer of final results.

The mRNA with pathways in $h^{\left(3\right)}$ was used as the data of the input layer, where each input node represents an mRNA gene. Each node in the pathway layer represents an independent biological pathway, and its connection with genes (that is, the upper layer) is obtained through the biological pathway database. There are only one or more genes on each pathway. Therefore, the subsequent analysis can also explain the model from the perspective of the pathway based on the pathway layer. However, the biological pathways do not play a role by themselves; biological systems include multiple interacting biological pathways so that the interaction between different paths can be expressed by connecting to the same node in the hidden layer, where the hidden layer represents the biological nonlinear association between paths. Finally, the posterior probability is calculated at the output layer. The output layer contains two nodes representing different results of the model. Through the continuous improvement of the accuracy of the model, the continuous changes in the network can be revealed. The following Equation 12 is the forward propagation formula of the PNN.

$$h^{\left(l+1\right)}=a\left(\left(W^{\left(l\right)}*P^{\left(l\right)}\right)h^{\left(l\right)}+b^{\left(l\right)}\right),$$ (12)

where *a* is the activation function, being RELU when l=3,4 and SOFTMAX when l=5 . *P* is a mask matrix which only works when l=3 . It is expressed as the connection between mRNA and pathway, which is predefined and will not change with the iteration of the neural network. * represents element-wise multiplication. *W* and *b* are the weight matrix and bias vector of the PNN, respectively.

For the imbalance of data, it is necessary to improve the cost function and use focal loss (Lin et al., 2020) in Equation 13 to solve this problem.

$$F=\frac{1}{K}\sum_{i=1}^{K}\mu_{i}{\left(1-{pt}_{i}\right)}^{\tau }c\left(y_{i},\tilde{y_{i}}\right)$$

$${loss}_{PNN}=F+\frac{1}{2}\lambda_{2}{\left\|W\right\|}_{2},$$ (13)

where $c\left(\cdot \right)$ represents the cross-entropy loss function, $y_{i}$ represents the label value of sample *i*, and $\tilde{y_{i}}$ represents the predict value of sample *i*; ${pt}_{i}$ represents the difficulty of sample *i*. If the predicted value is close to the real value, ${pt}_{i}$ is close to 1, which means that this sample is easy to predict and the weight is smaller, so the algorithm can pay more attention to the samples that are difficult to predict. τ is used to control the degree of ${pt}_{i}$ action, and $\mu_{i}$ is used to balance the data. *K* represents the number of samples, ${\left\|W\right\|}_{2}$ represents a $L^{2}-norm$ of *W*, and $\lambda_{2}$ is a regularization hyperparameter.

Therefore, according to the loss function formulas of the GNN and PNN, the final backpropagation formula is given as in Equation 14 follows.

$$W^{\left(l\right)}\leftarrow \left(1-\eta \lambda_{2}\right)W^{\left(l\right)}-\eta \frac{\partial F}{\partial W^{\left(l\right)}}-\eta \frac{\partial {loss}_{GNN}}{\partial W^{\left(l\right)}}$$

$$b^{\left(l\right)}\leftarrow b^{\left(l\right)}-\eta \frac{\partial F}{\partial b^{\left(l\right)}}-\eta \frac{\partial {loss}_{GNN}}{\partial b^{\left(l\right)}},$$ (14)

where η is a learning rate.

## 3 Results

### 3.1 Data source and preprocessing

Six diverse kinds of data were used in this study. The clinical prognosis data of LUSC was used as a predictive label to analyze the genetic relationship within lung cancer and its influence on prognosis. Multimodal data are involved in which $X_{1}$ stands for mRNA, $X_{2}$ stands for miRNA, and $X_{3}$ stands for lncRNA in the experiment. A total of 551 transcript data and 523 miRNA sequencing data were downloaded from the TCGA databases (https://portal.gdc.cancer.gov/), containing both health sample data and LUSC patient data. In order to maintain the consistency of data dimensions in the calculation process, 411 cases co-existing in the three kinds of data were selected. In addition, the downloaded data also include survival time and status. Patients who survived for more than 24 months were regarded as good prognosis samples (GP), and patients who died within 24 months were regarded as poor prognosis samples (PP), regardless of whether they survived later. Patients whose survival time was less than 24 months and who were still alive were excluded from the experimental data and were regarded as censored data. Finally, a total of 188 GP and 100 PP samples were obtained, of which PP patients accounted for about 35% of the samples, making this data unbalanced.

All human biological pathway data were extracted from the biological pathway data of the Molecular Signatures Database (MSigDB) (Liberzon et al., 2015). Only the data of biological pathways containing at least ten genes were kept because large pathways usually include small pathways. These genes are mRNA data after feature selection, and if there was biological pathway data, this gene was reserved as the input of the algorithm. After that, we constructed the biological pathway mask matrix P, in which the genes existing in the biological pathway are set to 1, or otherwise 0; a total of 428 fixed gene-biological pathway connections were obtained.

Human protein links information was obtained from the String Database (https://string-db.org). All links involving genes were screened out, obtaining a total of 38,284 items of PPI network data.

### 3.2 Construction of the ceRNA network

The mRNA and lncRNA data were first isolated from the transcript data. The Deseq2 R package developed by Love et al. (2014) was then used to analyze the differences between the three kinds of gene and identify the significant genes for subsequent processing to improve the accuracy of the algorithm. By using the thresholds of |logFC|>2 and *p*-value<0.05, 3347 characteristic mRNA expressions, 168 characteristic miRNA expressions, and 2,282 characteristic lncRNA expressions were obtained.

In order to determine the regulatory relationship between genes, miRNA data related to DElncRNA were identified from the “mircode” database (Jeggari et al., 2012) to obtain the miRNA–lncRNA relationship pair. Then, the “starbase” database (Li et al., 2014) was used to label miRNA with 3P and 5P to find the target mRNA of miRNA in the miRDB (Wong and Wang, 2015), miRTarBase (Chou et al., 2016), and TargetScan (Garcia et al., 2011) databases. Lastly, their intersections were obtained, and miRNA–mRNA relationship pairs were obtained to construct ceRNA networks.

### 3.3 Experimental setting

Following the design idea of the neural network, its parameters were determined and adjusted by repeated experiments for accuracy as the reference standard before random validation. The learning rate was η = 0.0001, λ_1 = 0.1, and λ_2 = 0.0004. The adaptive moment estimation (“Adam”) was used as the optimizer (Kingma and Ba, 2014). Each training had a batch size of 64, with a total of 350 iterations. In addition, the number of nodes in each layer of the neural network needed to be consistent with the actual number to ensure the interpretability of the model. For example, the biological pathway layer had 1,104 nodes because there were 1,104 pathways in total. The hidden layer had 500 nodes, which was the best parameter obtained by increasing the number of nodes in the experiment. Finally, using pyTorch as a tool to build a neural network framework, DGL was used to build the GNN. The source code is available at https://github.com/safu-HL/ISGPN, and the result can be copied.

### 3.4 Result and comparison

Through five repetitions of ten-fold random validation, the average accuracy of TSGPN was 68.2758%, of which the verification and the test sets accounted for 10% each, and the training set accounting for 80%. In order to further evaluate the performance of TSGPN, we compared it with the support vector machine (SVM), random forest (RF), and Lasso logistic regression (LLR). We also compared this algorithm without feature selection (N-TSGPN) to show the importance of feature selection. These algorithms were repeatedly calculated to obtain the best parameters. For the reproducibility of the results, the calculated results were obtained by ten-fold random validation. The average and standard deviation of the data on each fold were calculated to standardize the data.

**FIGURE 2:**
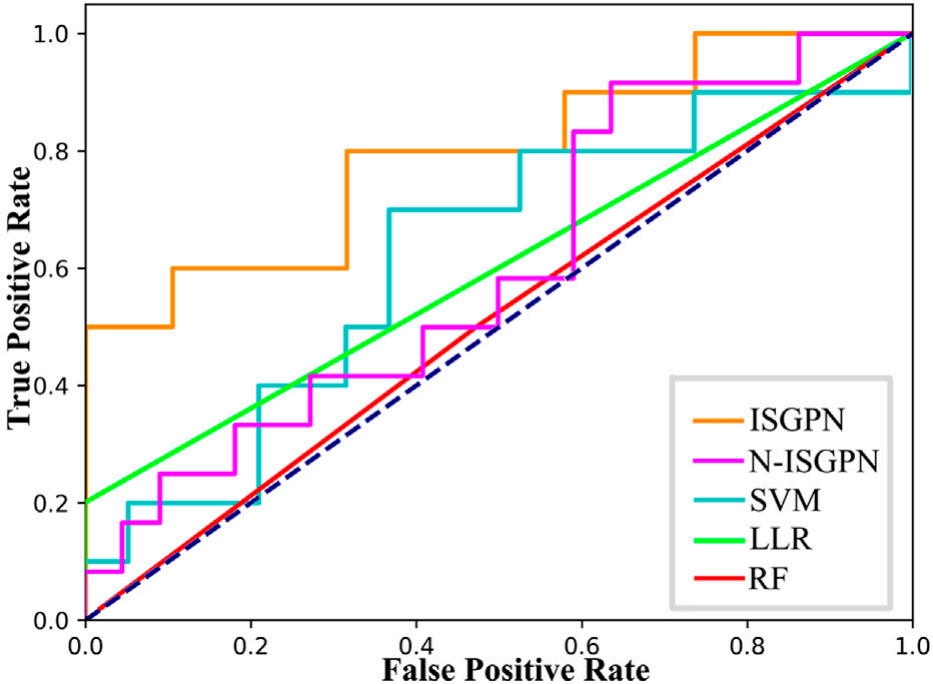
ROC curves of each algorithm.

For N-TSGPN, all involved genes were added to GNN, and the weights of the edges were distributed by matrix operation. Specifically, each batch of data $X=\left[X_{1},X_{2},...,X_{lnc},X_{lnc+1},...,X_{mi},X_{mi+1},...,X_{m}\right]$ contained vectors of lncRNA, miRNA, and mRNA, respectively. We multiplied lncRNA with miRNA and miRNA with mRNA and performed SoftMax on each column to determine the weight of each edge between different genes; the sum of the weight values was 1. This is obviously similar to a fully connected network. Thence, the prediction performance of this algorithm was evaluated by using the area under the curve (AUC) and F1-scores. The receiver operating characteristic (ROC) curve is drawn in Figure 2 to check the accuracy of the details of the algorithms. The following indexes in Equations 15–18 are usually used to compare the performance of several prediction models.

$$Accuracy=\frac{TP+TN}{TP+TN+FP+FN,}$$ (15)

$$Recall=Sensitivity=\frac{TP}{TP+FN},$$ (16)

$$Specificity=\frac{TN}{TN+FP,}$$ (17)

$$Precision=\frac{TP}{TP+FP},$$ (18)

The results of algorithm comparison and calculation are shown in Table 1. It can be seen from there that the proposed TSGPN method is superior to other algorithms in average AUC and F1-score. The top algorithms are all nonlinear, which indicates that the relationship between genes requires deeper mining; the model that can capture the nonlinear relationship will have more advantages. Compared with N-TSGPN, the accuracy of TSGPN with feature selection has been greatly improved, ensuring the sparsity and interpretability of the algorithm and also demonstrating the importance of feature selection. Moreover, the accuracy of cancer prognosis prediction is generally not very high. For example, Zhang et al. (2023) used a local augmented GNN (LAGProg) to enhance the model’s ability to express multi-omics characteristics. This extracts the features in the omics data and biological network that meet the enhancement conditions and then feeds the enhanced features and original features back to the prognosis prediction model. By verifying different data sets, it was concluded that the C-index values of the model with LAGProg increased by 8.5% on average, but the accuracy of most data was still between 0.6 and 0.8, and the accuracy of LUSC was 0.625.

**TABLE 1:** Comparison of AUC and F1-SCORE.

Model	AUC	F1-score
TSGPN	68.2758	0.4505
N-TSGPN	61.7641	0.1333
SVM	65.5172	0
LLR	52.4138	0.3268
RF	64.1379	0.1725
LAGProg	62.5	0

In order to illustrate the performance difference between TSGPN and other algorithms, the Wilcoxon signed-rank test, a non-parametric paired bilateral test, was used in this study. First, it is assumed that TSGPN is not much different from other methods ( $H_{0}$ ). Then, the predicted values after the algorithm operation were used as data and were tested with the predicted values of TSGPN respectively using the Wilcoxon signed-rank test. As shown in Table 2, TSGPN rejects the original hypothesis $H_{0}$ at the significance level of 5% (*p*-value<0.05)—the performance of TSGPN is obviously superior to other algorithms, which is statistically significant.

**TABLE 2:** Wilcoxon signed-rank tests.

	W statistic	*p*-value
TSGPN vs SVM	6.5393	6.18e-11
TSGPN vs LLR	3.8334	1.26e-04
TSGPN vs RF	5.6373	1.73e-08

## 4 Discussion

In this section, the results of the algorithm are fully explained and visualized. Because the meaning of each node is defined at the beginning of designing the model, every step in the algorithm can be explained. According to the sequence of the algorithm, the strong and weak connections between key biomolecular markers, the possibility of genes corresponding to biological pathways, and the mutual combination of pathways are displayed. We use particular analyses to obtain the possible reasons and explanations of the results of the algorithm, such as a key biomolecule marker, cell activity in biological pathways, or their combination.

### 4.1 Prediction, reconstruction, and analysis of ceRNA network

**FIGURE 3:**
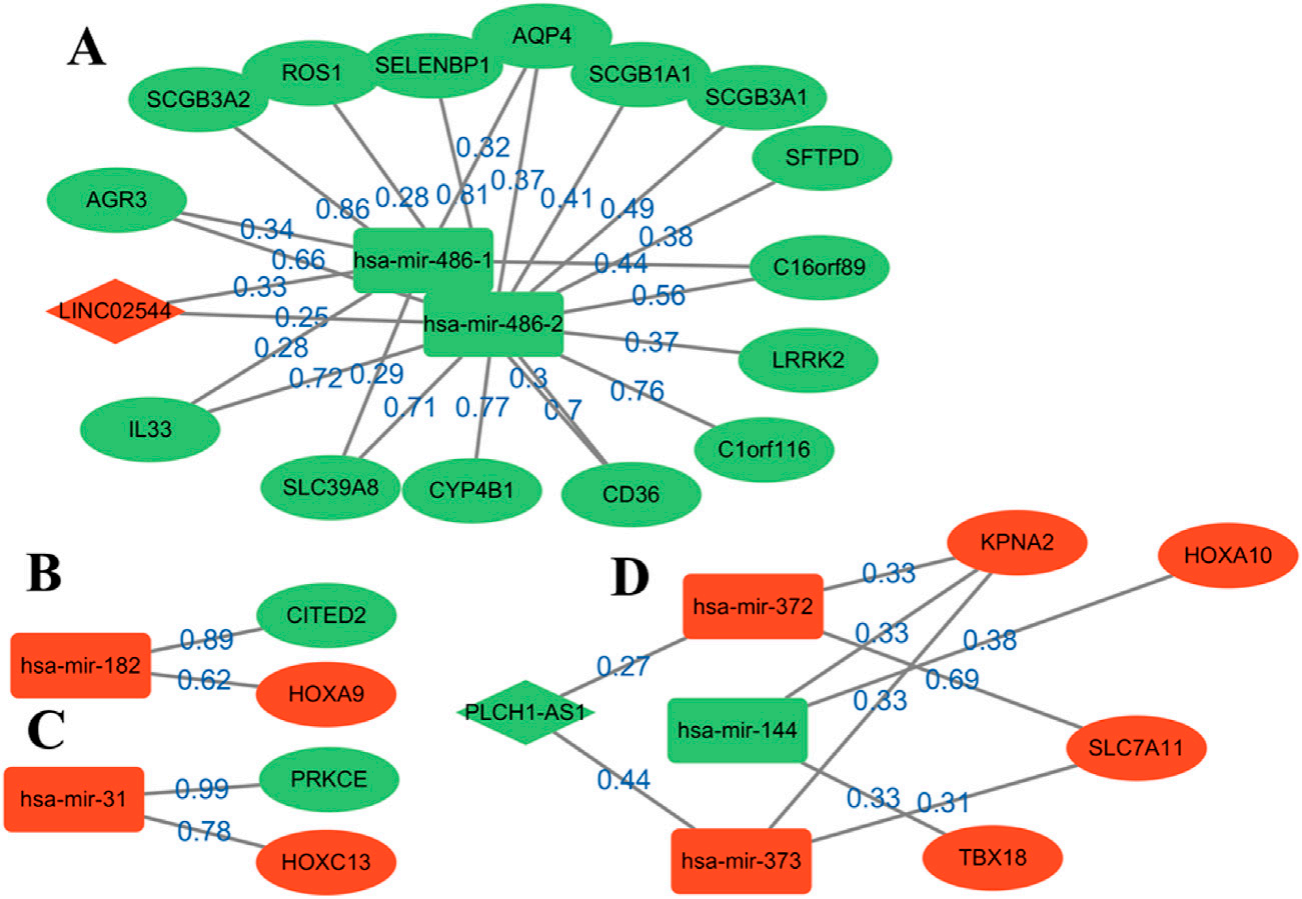
Reconstruction diagram of the ceRNA network based on GNN prediction. Most of the weights are over 0.6. Oval nodes represent mRNAs; square nodes represent miRNAs; diamond nodes represent lncRNAs. Red/green indicates that the gene is up-/downregulated in the patient for healthy, and weight represents the strength of the relationship between them. A total of four modules, **(A–D)**, are obtained for the following analysis.

Through the continuous iterations of the model, the sparse network with the accurate values of connections between nodes is obtained. The number of edges dropped from 842 to 340, which greatly helped identify and explain important edges. The top edges are sorted according to the weight values (Figure 3). By analyzing the up- and downregulated genes in the network and the connection trend of genes, the possible key biomarkers of LUSC prognosis can be predicted.

Four modules are obtained, two of which have a complete ceRNA network, while the other two lack lncRNA. An extensive literature search found that most of the genes in Figure 3 have different effects on the prognosis of lung cancer. Using this evidence, other genes in the algorithm results are more likely to be verified by biologists, which provides a reference for the key biomarkers of LUSC prognosis. Module A contains the LINC02544 lncRNA, the upregulation of which enhances the proliferation, migration, and invasion of LUSC cells (Wei et al., 2022). Therefore, it may be possible to obtain the biomarkers for the prognosis or target treatment of LUSC by identifying the downregulated genes associated with it in Figure 3. In particular, the weights of LINC02544 and hsa-mir-486-1/hsa-mir-486-2 are 0.33 and 0.25 respectively, in which hsa-mir-486-1 and–2 belong to the same miRNA family. These can affect mRNA stability and translation to regulate gene expression after transcription in multicellular organisms. The TGF-beta signal can induce EMT and plays an important role in it. The overexpression of hsa-mir-486 can suppress TGF-beta-induced EMT as well as the migration and invasion of NSCLC cells (Chen et al., 2019). The low expression of hsa-mir-486 in LUSC may be used as an anti-cancer gene and is an important biomarker. The overexpression of SELENBP1 is obviously related to hsa-mir-468-2, showing an inhibitory effect on NSCLC (Zhu et al., 2023). More importantly, the downregulation of SELENBP1 is an early event of LUSC, which increases bronchial epithelial cell transformation and may be used as a new potential biomarker for the early detection of LUSC (Zeng et al., 2013). In addition, it was found that C1orf116 has a high weight and is closely related to the EMT process, which may be a key early event in tumor metastasis. The low expression of C1orf116 is related to the poor prognosis of lung cancer (Parsana et al., 2017).

In module D, the intricate gene relationship in the ceRNA network is well displayed. Ma et al. (2019) identified the relationship between hsa-mir-144 and HOXA10, which proved the reliability of the proposed algorithm. They found that HOXA10 would be downregulated in response to the overexpression of hsa-mir-144, while inhibiting LINC00466 would reduce its binding with hsa-mir-144, thus upregulating the latter. The upregulation of hsa-mir-144 and downregulation of HOXA10 exert an inhibitory role in tumorigenicity, invasion, migration, and proliferation and also promote the apoptosis of LUAD cells. Therefore, hsa-mir-144 is a potential biomarker, and LINC00466 still exists in the remaining connections, although it does not appear in module D. In addition, the overexpression of HOXA10 is closely related to the clinical stage of LUSC, which plays a key role in non-small cell carcinoma; this effect is more obvious in LUSC than in LUAD (Guo et al., 2019). At the same time, other genes in the module also show effects on lung cancer, such as KPNA2 and SLC7A11, and their overexpression can promote the growth of cancer cells (Zhou et al., 2017; Liu et al., 2020).

Moreover, several pairs of mRNA–miRNA relationships with greater weight were found. For example, Luo et al. (2018) found that hsa-mir-182 played an important role in LUSC and revealed the molecular mechanism of LUSC through the PPI network, GO, and KEGG enrichment analysis. Among these, PRKCE is located in the center of the PPI network, which has a strong influence on molecular mechanism. Tan et al. (2011) identified a 5-mircroRNA classifier to distinguish LUSC and normal tissues, including hsa-mir-182 and hsa-mir-486. The high expression of hsa-mir-31 was also related to the low survival rate of LUSC. This showed that these genes play a representative role in LUSC. On the whole, determining the relationship in the ceRNA network through GNNs can help us analyze the interaction between genes and find potential biomarkers. The above analysis also demonstrates the accuracy of the results and provides a possible scheme for the search for targeted genes.

### 4.2 Analysis of LUSC based on the biological pathway

Through the GNN network, we can identify the key mRNA genes according to their weights and then analyze the internal mechanism of LUSC in terms of molecular pathways and genes. For each biological pathway, the absolute values of weights of the key genes are calculated and added, and the top ten are selected as the main pathways.

**FIGURE 4:**
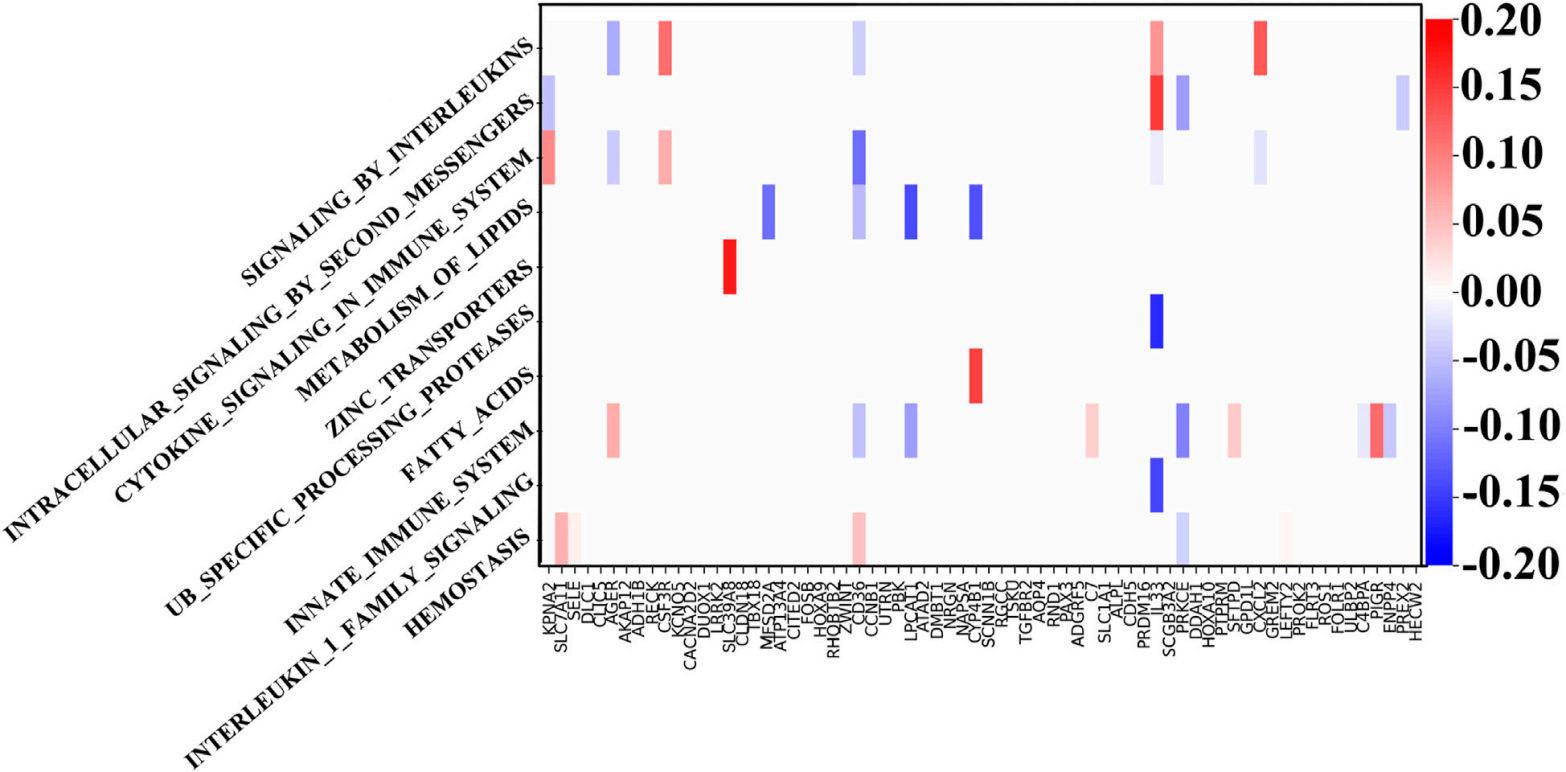
Weights between genes and ten top-ranked pathways. The key genes of each pathway are determined by the biological pathway database, in which the sum of absolute values of the key gene weights is the largest in the first pathway.

Figure 4 shows the weight between biological pathway and gene. The first pathway from top to bottom has the most obvious relationship with genes and is arranged in descending order. The “REACTOME SIGNALING BY INTERLEUKINS” pathway corresponds to IL33, which shows good diagnostic performance for NSCLC (Hu et al., 2013). Similarly, the expression of SLC39A4 is significantly correlated with tumor size and overall survival in the “REACTOME ZINC TRANSPORTERS” pathway (Wu et al., 2017). SLC39A8 and SLC39A4 are both a kind of zinc transporter. In addition, the content of diacylglycerol in the “REACTOME INTRACELLULAR SIGNALING BY SECOND MESSENGERS” pathway can be used as a biomarker for the early detection and prognosis monitoring of LUSC (Casamassima et al., 1996). Interestingly, LUSC is more dependent on glucose than LUAD in the “FATTY ACIDS” pathway, and the genes related to the fatty acid metabolism have also increased (Leitner et al., 2022). LUAD’s metabolism is more flexible, and metabolic adjunct therapy may be more successful in LUSC than LUAD. Genes related to the “METABOLISM OF LIPIDS” pathway are PTGIS and HRASLS, and they are also related to tumor immunity (Lei et al., 2023). Another metabolite, adenosine diphosphate, is helpful for hemostasis, angiogenesis, cell proliferation, and metastasis, which occurs in the HEMOSTASIS pathway and may be a potential therapeutic target (Hoang et al., 2019). In summary, the pathways related to LUSC include interleukin, protein cells, lipid metabolism, and immune system pathways.

**FIGURE 5:**
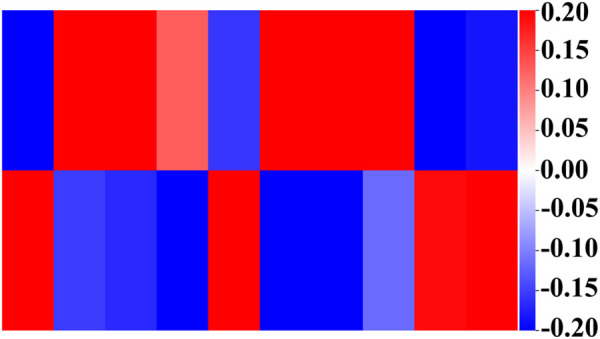
Weights of the output layer with the ten top-ranked hidden nodes. The upper layer represents the output with good prognosis, and the lower layer represents the output with poor prognosis. Their proportion is consistent with the proportion of overall data.

Next, we show the weights in the hidden and output layers in Figure 5 revealing the different weight patterns of the two output neurons. We select the top ten hidden layer nodes with the largest output difference, which represents the most obvious pathway combination. Moreover, the positive and negative sample outputs of these ten nodes are also in proportion to the data.

**FIGURE 6:**
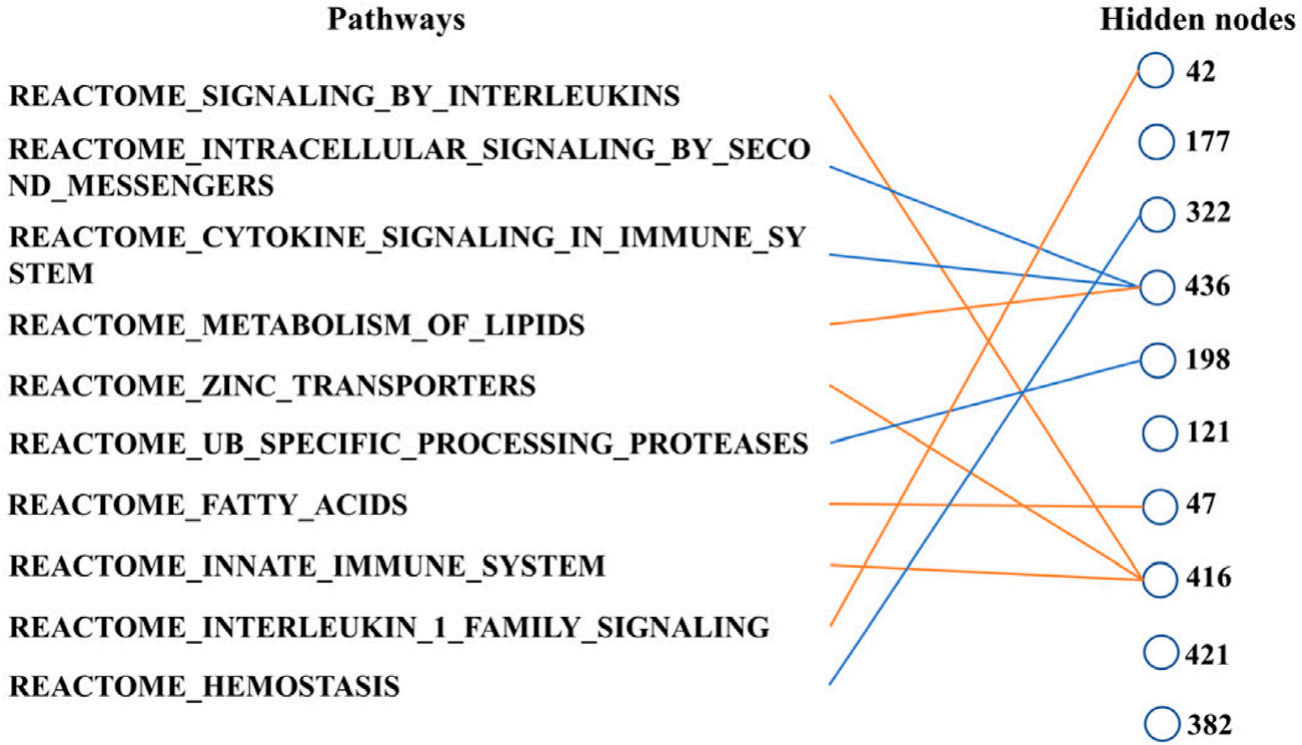
Main connections between pathway layer nodes and hidden layer nodes. Blue line represents negative weight, and the orange line represents positive weight. Pathways and hidden layer nodes are in the ten top-ranked.

Finally, we can determine the relationship between the pathways according to Figure 6. These ten biological pathways simply correspond to the mRNA gene in the previous step—the top ten biological pathways with the greatest weight. Similarly, the top ten hidden layer nodes with the greatest weight are selected to represent the interaction of these paths. The calculation of node weight is the sum of the absolute value of all weights. Then, for each hidden layer node, we select the pathway layer node with the largest absolute weight among all nodes connected to this hidden layer node. As shown in Figure 6, hidden layer nodes 436 and 416 aggregate multiple paths, meaning that there may be mutual restriction or promotion between them for subsequent analysis. The innate immune system, zinc transporters, and signaling by interleukins may have a positive relationship, as well as intracellular signaling by second messengers and cytokine signaling in the immune system. However, the metabolism of lipids seems to have a negative relationship with them.

## 5 Conclusion

The TSGPN method proposed here reveals the interaction between genes very well. The expected result is not only to predict the prognosis of LUSC, but more importantly, to determine the key pathogenic factors and their potential correlation using the transparency of the model. First, according to the simulation of the biological network, a fully connected graph neural network is constructed, and the hard concrete gradient estimator is used to gradually make the connections between networks sparser during the training process. At the same time, prior knowledge of the ceRNA and PPI networks is added to make the results more accurate. After obtaining the reconstructed ceRNA network with weight values, the pathway database is added to make the results of the algorithm more bio-interpretable, and its activities in organisms can be analyzed from the perspective of pathways. Through the continuous improvement of the prediction accuracy of the model, the internal network of the model is also changing, and this is transparent. Because every node in the network has a defined meaning, we can predict the possible connections between genes or between them and pathways according to the experimental results. These results showed that TSGPN is superior to SVM, RF, and LLR in the AUC and F1 scores, and the feature selection is beneficial for improving the accuracy of the algorithm.

## Data Availability

The gene and clinical data presented in the study are deposited in the TCGA repository, accession website https://portal.gdc.cancer.gov/. The bio-pathway data presented in the study are deposited in the MSigDB repository, accession website https://www.gsea-msigdb.org/gsea/msigdb. The PPI data presented in the study are deposited in the String repository, accession website https://string-db.org.
